# Genetic Analysis of the Red Anther Trait Introgressed into Upland Cotton (*Gossypium hirsutum*) from *Gossypium armourianum*

**DOI:** 10.3390/plants15162544

**Published:** 2026-08-21

**Authors:** Jinfa Zhang, James McDonald Stewart

**Affiliations:** 1 School of Agriculture, Tennessee Tech University, Cookeville, TN 38505, USA; 2 Department of Crop, Soil and Environmental Sciences, University of Arkansas, Fayetteville, AR 72701, USA

**Keywords:** cotton, introgression, *Gossypium hirsutum*, *Gossypium armourianum*, red anther

## Abstract

Upland cotton (*Gossypium hirsutum*, AD1) is the most important fiber crop for the world’s textile industry. The genetic base of Upland cotton is narrow, and the introgression of germplasm from other species into Upland cotton can broaden its genetic diversity, including the introduction of new qualitative traits. The objectives of this study were to determine the inheritance, allelism, and linkage relationships of the red anther trait introduced into Upland from the red anther donor *G. armourianum* (D2-1) through crosses involving a synthetic tetraploid A1D2-1. After two generations of backcrossing to Upland cotton, followed by inbreeding, homozygous red-anthered plants (RA) were selected and used for crosses with two Upland lines, the Upland standard TM-1, the multiple recessive marker line T582, and the multiple dominant marker line T586. All Upland cotton parents, except the RA parents and T586 (possessing yellow anthers), had cream anthers. The anther color of the F*_1_* plants was red, indicating that the red anther trait is dominant. In nine of ten F*_2_* populations and four of five testcross populations, the segregation of anther color followed a 3:1 and 1:1 ratio, respectively, as expected under a one-gene model. In three F*_2_* populations with a total of 885 plants between RA with green leaf and T586 with red leaf (*R_1_* on chromosome D07), no double recessive genotype (yellow/cream anther and green leaf) was observed, indicating the allelic relationship between *R_1_* and red anther allele, designated *R_1_^a^*. The recombination frequency between *R_1_^a^* and cluster-1 (*cl_1_*) on D07 was estimated to be 0.112 in 256 F*_2_* plants of a cross between RA and T582 based on the maximum likelihood estimation. This new red anther trait and its associated new gene allele provide an important genetic source for understanding the biosynthesis of anthocyanins and may have utility for improving resistance to anther-feeding insects in cotton.

## 1. Introduction

Cotton (*Gossypium* spp.) is the world’s most important natural fiber crop for the textile industry, and it is grown in more than 80 countries [1]. Tetraploid Upland cotton (*G. hirsutum*, 2n = 4x = 52, AD1) accounts for approximately 97% of the world cotton production, while the remainder is from another tetraploid *G. barbadense* (2n = 4x = 52, AD2), known as extra-long staple, Sea-Island, Egyptian, or Pima cotton. Cotton production faces numerous challenges which require solutions via genetics and breeding.

Breeding has continued to improve yield and other traits in Upland cotton [2,3,4]. However, the narrow genetic base and variation within Upland cotton remain major concerns. The introgression of novel germplasm, traits and genes from other *Gossypium* species into Upland cotton provides an important avenue to enhancing the genetic diversity of Upland cotton [5]. The two cultivated tetraploid cotton species evolved from the same allotetraploid ancestor approximately 1–2 million years ago through a natural hybridization event between an Old-World A-genome and a New World D-genome diploid, and they are cross compatible. Thus, extensive efforts in directly crossing and introducing germplasm from *G. barbadense* to Upland cotton have been made by numerous cotton researchers since the rediscovery of the Mendelian hereditary law in 1900, but these have met only limited success [6,7].

In addition to the two cultivated tetraploid species, there are three wild tetraploid species of the same origin as *G. hirsutum* and *G. barbadense* and more than 40 diploid species (2n = 2x = 26) in the *Gossypium* genus [8]. The diploid species are classified into eight genomic groups, i.e., A, B, C, D, E, F, G and K [5]. There are several approaches to introducing germplasm from these diploid species into Upland cotton, and the most efficient method is through the synthetic tetraploid pathway of a hybrid between an A genome and a D genome species [5]. Through this pathway, several trispecies populations (e.g., A2D8AD1 and A1D2-1AD1) have been produced [9,10].

In cotton, more than 200 major genes and alleles for qualitative traits have been identified [11,12,13] from various sources, including natural mutants or mutants found in breeding nurseries/fields or induced, or new traits introduced from other species [12]. Morphological mutants are useful genetic markers in the study of basic cotton plant biology in growth and development. Some of them are also useful in cotton breeding, especially those that were introduced into Upland cotton from other species, such as nectariless genes *ne_1_ne_2_* from *G. tomentosum* [14] for insect resistance [15], and bacterial blight resistance genes *B_4_* and *B_6_* from *G. arboreum*, *B_5_* from *G. barbadense*, *B_8_* from *G. anomalum*, and *B_9K_* and *B_11_* from *G. herbaceum* [16].

*G. armourianum* (D2-1) is a wild diploid species, native to the arid, semi-arid, and coastal regions of northwestern Mexico [17]. It is tolerant to drought and resistant to several insects and diseases such as jassids, thrips, whiteflies, the tarnished plant bug, and cotton leaf curl disease [18,19,20]. It also possesses red anthers and petal spot [17] (see https://www.cottongen.org/organism/Gossypium/armourianum, accessed on 18 August 2026). A triploid hybrid between *G. hrisutum* and *G. armourianum* was made in India [18], which was followed by direct backcrossing to *G. hirsutum* for two generations [19,20]. In the early 1990s, James McD. Stewart, at the University of Arkansas, produced a synthetic interspecific tetraploid between *G. herbaceum* (A1) and *G. armourianum*, which was then backcrossed to cream-anthered *G. hirsutum* twice [10]. The objective of this study was to report the genetic basis of the red anther trait after it was transferred into Upland cotton. This new trait and new gene allele will provide an important genetic source to understand the biosynthesis of anthocyanins and may have potential applications in breeding for enhanced resistance to anther-feeding insects in cotton.

## 2. Results and Discussion

**Figure 1:**
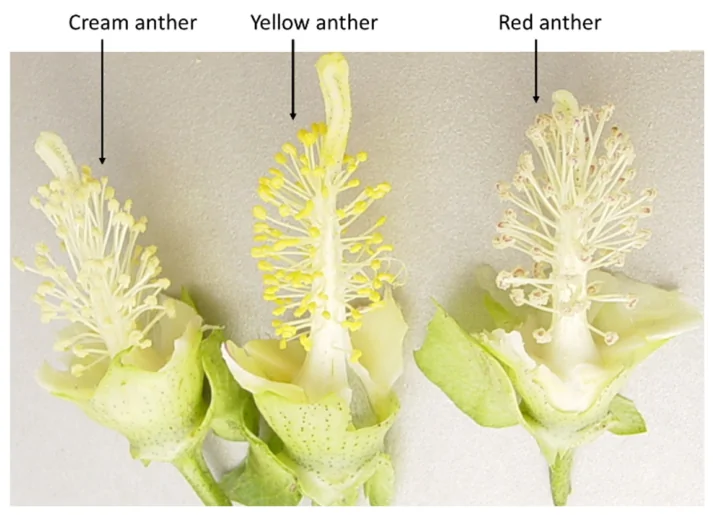
Anther colors in Upland cotton (*Gossypium hirsutum*): cream anthers and cream pollen in most Upland cotton lines; yellow anthers and yellow pollen in T586 and *Gossypium barbadense*; red anthers and cream pollen transferred from *Gossypium armourianum*.

All Upland cotton parents except the RA parents and T586 (with yellow anthers) had cream anthers. At 1–3 days before anthesis, the anther color in large floral buds is fully developed. Anther color can also be distinctly observed at flowering (Figure 1). Anther color in all F*_1_* plants was red, indicating that the red anther trait is dominant.

A segregating ratio between plants with red anthers and cream anthers in F*_2_* and testcross populations should indicate the genetic basis of the red anther trait. Except for one F*_2_* population between T586 (the female parent) and RA, the segregation for anther color in all 10 F*_2_* populations followed a 3:1 ratio (Table 1), as expected for a single dominant gene controlling red anther color. For the (T586 × RA)F*_2_* population, the number of non-red-anthered plants was higher than expected (0.01 < *p* < 0.05). However, its reciprocal F*_2_* population followed a 3:1 ratio, suggesting the possible presence of a low percentage of false hybrid seeds when the F*_1_* cross was made using T586 as female. We should point out that in red-anthered parents, pollen had a cream color. In T586, however, the yellow pollen gene *P_1_* also confers yellow color to anthers. In the (RA × T586)F*_2_* population, the ¼ of non-red anthered plants had either yellow or cream anthers/pollen.

Similarly, the number of recessive cream-anthered plants was also unexpectedly higher (*p* < 0.01) in the initial testcross between (RA × ST 506)F*_1_* and ST 506 (Table 2). However, segregation for anther color in four other testcrosses with TM-1 or T586 as the testcross parent all fit the expected 1:1 ratio based on a single-gene model (Table 2). As expected, all F*_1_* plants from crosses with RA had red-anthers, further confirming the dominant inheritance of the red anther trait.

Interestingly, the testcross with T-586 (with yellow anther and pollen) as the testcross parent also showed 1 red:1 yellow ratio, suggesting that the red anther trait is also dominant to yellow anther. The yellow anther/pollen trait is controlled by a dominant gene, *P_1_*, on chromosome A05 [11], and the cream anthers/pollen (*p_1_*) is recessive to yellow anthers/pollen. However, whether the red anther gene is allelic to *P_1_* remained to be determined.

T586 possesses *R_1_* (red plant) on chromosome D07 and *R_2_* (red petal spot) on A07. In (RA × T586)F*_2_*, segregation between the red anther trait and all the marker genes except *R_1_* showed independent assortment, suggesting that the red anther gene is not linked to eight of the nine marker genes, including *P_1_* and *R_2_*. However, among 715 F*_2_* plants, no individuals exhibited both cream anthers and green plants (i.e., the double recessive phenotype), which would result from genetic recombination between the red anther–green leaf parent and the cream anther–red plant (T586). Similar results were observed in two additional F*_2_* populations in which T586 was crossed with two homozygous red anther lines (Table 3). The goodness-of-fit testing results for these three F*_2_* populations showed that the two traits (red anther and red plant) are not independently inherited. Instead, they are co-segregated or completely linked.

We then conducted an allelic test between the red anther and red plant genes. Results from both F*_2_* populations showed that the segregation followed a 1 (red anther–green leaf):2 (red anther–red leaf):1 (cream anther–red leaf) ratio (Table 4), suggesting that the red anther gene and red leaf *R_1_* genes are allelic. Therefore, the red anther allele is designated *R_1_^a^* on chromosome D07.

Among the five recessive gene markers in T582, the short fruiting branch (cluster-1) gene, *cl_1_*, is located on the same chromosome D07 as *R_1_*. We then tested if *R_1_^a^* and *cl_1_* were independently assorted in (RA × T582)F*_2_*, and the results showed that the two genes were linked rather than independently assorted (Table 5). Based on maximum likelihood estimation, the estimated recombination frequency was 0.112, with a standard error of 0.021. The corresponding genetic distance was 12.7 cM based on Haldane’s mapping function, which is lower than the reported genetic distance of 17 cM between *R_1_* and *cl_1_* [11,12]. Estimated recombination frequencies (and corresponding genetic distances) can vary depending on the cross combination.

Because no recombinants between *R_1_^a^* and *R_1_* were observed in the (RA × T586)F*_2_* population of 885 plants, the difference in estimated genetic distances may be due to suppressed recombination in the genomic region containing *R_1_*/*R_1_^a^* between the homologous chromosomes of D07 from Upland cotton and *G. armourianum*. Sequence and cryptic structure differences between the two genomes may suppress recombination in this region. In fact, it is well known that cryptic genome structural differences, such as minor chromosomal inversions, translocations, deletions, duplications, and asymmetric divergence between genomes or subgenomes in cotton, suppress genetic recombination between homologous chromosomes in interspecific hybrids, as evidenced in crosses between *G. hirsutum* and *G. barbadense* [21].

## 3. Conclusions

The red anther trait was transferred into Upland cotton from *G. armourianum* through crossing with a synthetic tetraploid A1D2-1 and subsequent backcrossing to Upland cotton. The detailed genetic analysis in this study showed that the red anther trait is conditioned by a dominant gene *R_1_^a^*, which is allelic to the red plant gene *R_1_* on chromosome D07, with a genetic recombination of 0.112 from the cluster-1 gene *cl_1_*. Red (anthocyanin) pigmentation in cotton occurs in various plant organs and tissues, including leaves, veins, stems, calyx, petals, petal spot, anthers, anther filaments, and even fibers. The genes controlling red pigmentation, including *R_1_* (red plant) on D07 [22], *Re* (red leaf) also on D07 [23], *Rs* (sub-red plant) on A07 [24], and *DR* (dwarf red) on A09 [25], have recently been cloned and characterized [13]. The red anther *R_1_^a^* allele can be isolated and sequenced through PCR, sequencing, and chromosome walking (if needed) based on the *R_1_* gene sequence of Upland cotton and the sequenced genome of *G. armourianum*. This new red anther trait and the novel allele *R_1_^a^* provide an important genetic resource for understanding the regulations of anthocyanin biosynthesis [26,27] and may have practical applications in cotton breeding, particularly for enhancing resistance to anther-feeding insects in Upland cotton [28,29].

## 4. Materials and Methods

### 4.1. Transfer of Red Anther to Upland Cotton

An A1D2-1 diploid hybrid was initially produced by crossing a *G. herbaceum* (A1 genome) accession as the female parent with a *G. armourianum* (D2-1 genome) accession using ovule culture techniques [30]. The diploid AD hybrid was then treated with colchicine to produce a synthetic allotetraploid A1A1D2-1D2-1. The synthetic tetraploid was then backcrossed to Upland cotton for two generations, followed by self-pollination to generate a segregating population, in which plants with red anthers were identified [10].

### 4.2. Parents, F_1_, and Segregating Populations from Different Cross Combinations

Dark red anthered plants homozygous for the anther color (designated as RA) were selected from the segregating population and crossed with five Upland genotypes: commercial cultivar ST 506; an Upland line designated as AD1 of unknown pedigree; Upland cotton genetic standard TM-1 [31]; the multiple recessive marker line T582 [11,32]; and the multiple dominant marker line T586 [11,32]. For each cross combination, reciprocal crosses were made. The T582 line possesses the following five recessive marker loci: virescent-1, *v_1_*, on chromosome 20 (D10); cup leaf, *cu*, location unknown; glandless-1, *gl_1_*, location unknown; frego bract, *fg*, on chromosome 3 (A03); and cluster-1, *cl_1_*, on chromosome 16 (D07). The T586 line carries 9 dominant marker loci: red plant, *R_1_*, on chromosome 16 (D07); okra leaf, *L_2_^o^*, on chromosome 15 (D01); tomentum, *T_1_*, on chromosome 6 (A06); red petal spot, *R_2_*, on chromosome 7 (A07); yellow pollen, *P_1_*, on chromosome 5 (A05); yellow petals, *Y_1_*, on chromosome 13 (A13); brown lint, *Lc_1_*, on chromosome 7 (A07); green lint, *Lg*, on chromosome 15 (D01); and naked seed, *N_1_*, on chromosome 12 (A12) [11,12]. Many of these genes have been cloned, and their underlying candidate genes have been identified [13,33]. The resulting F*_1_* plants were self-pollinated to produce F*_2_* populations and were also backcrossed (or testcrossed) to the parental line for segregation and linkage analyses.

Due to unexpected segregation ratios, single plant selections for red anthers were made from the initial segregating population to produce two homozygous red anther progeny B24-3RA and B24-4RA. The two RA lines were further used to cross with TM-1 and T586 to generate F*_1_*, F*_2_* and testcross (backcross) generations for segregation and linkage analyses.

### 4.3. Evaluation of Anther Color and Statistical Analysis

All plants, including the parents, were grown under field conditions. In red-anther plants, the red pigment in the anther wall becomes visible when the floral bud is between 3 and 4 mm in length and continues to intensify as the bud develops. At the flowering stage, anther colors (red, cream, or yellow) were evaluated for each plant using larger floral buds, typically 1–3 days before anthesis, without ambiguity.

The Chi-square (χ^2^) test of goodness-of-fit was used to determine whether anther color followed expected Mendelian segregation ratios (3:1 in an F*_2_*, 1:1 in a testcross, and 9:3:3:1 for two-gene segregation in an F*_2_*). Maximum likelihood estimation [34] was used to calculate recombination frequency between red anther (*R_1_^a^*) and cluster (*cl_1_*) in (RA × T582)F*_2_*. To convert recombination frequency to mapping distance, Haldane’s mapping function [34] was used. All the statistical analyses were performed using Excel. Results from reciprocal crosses of the same cross combination were combined when no significant differences were observed between the reciprocal crosses.

## Figures and Tables

**Table 1:** Inheritance of red anther transferred from *Gossypium armourianum* to Upland cotton (*G. hirsutum*): F2 generation.

Cross	Red Anther	Cream Anther	Expected Ratio	Chi-Square
(RA × ST 506)F*_2_*	138	42	3:1	0.27
(RA × TM-1)F*_2_*	179	58	3:1	0.04
(RA × T582)F*_2_*	234	96	3:1	2.95
(RA × T586)F*_2_*	256	96 ^a^	3:1	0.97
(T586 × RA)F*_2_*	254	109 ^a^	3:1	4.75 *
(RA × AD1)F*_2_*	109	41	3:1	0.44
(B24-4RA × TM-1)F*_2_*	62	24	3:1	0.39
(B24-3RA × TM-1)F*_2_*	129	42	3:1	0.02
(B24-4RA × T586)F*_2_*	55	19 ^a^	3:1	0.02
(B24-3RA × T586)F*_2_*	76	20 ^a^	3:1	0.89

* *p* < 0.05. ^a^ yellow or cream anthers/pollen due to segregation of the *P_1_* gene.

**Table 2:** Inheritance of red anther transferred from *Gossypium armourianum* to Upland cotton (*G. hirsutum*): Testcrosses.

Cross	Red Anther	Cream Anther	Expected Ratio	Chi-Square
(RA × ST 506)F*_1_* × ST 506	97	151	1:1	11.76 **
(RA × TM-1)F*_1_* × TM-1	35	33	1:1	0.06
(RA × T586)F*_1_* × T586	86	106 ^a^	1:1	2.08
(B24-4RA × TM-1)F*_1_* × TM-1	16	22	1:1	0.95
(B24-3RA × TM-1)F*_1_* × TM-1	18	12	1:1	1.20

** *p* < 0.01. ^a^ yellow anther.

**Table 3:** Test for independent assortment between red anther and red leaf traits in Upland cotton (*Gossypium hirsutum*).

Cross	Red Anther, Red Leaf	Red Anther, Green Leaf	Cream or Yellow Anther, Red Leaf	Cream or Yellow Anther, Green Leaf	Expected Ratio	Chi-Square
(B24-4RA × T586)F*_2_*	34	21	19	0	9:3:3:1	11.57 **
(B24-3RA × T586)F*_2_*	52	20	24	0	9:3:3:1	8.30 *

* *p* < 0.05. ** *p* < 0.01.

**Table 4:** Allelic test between red anther and red leaf (*R_1_*) in Upland cotton (*Gossypium hirsutum*).

Cross	Red Anther, Green Leaf	Red Anther, Red Leaf	Cream or Yellow Anther, Red Leaf	Expected Ratio	Chi-Square
(B24-4RA × T586)F*_2_*	21	34	19	1:2:1	0.59
(B24-3RA × T586)F*_2_*	24	52	20	1:2:1	1.00

**Table 5:** Independent assortment between red anther (*R_1_^a^*) and cluster (*cl_1_*) in T582 of Upland cotton (*Gossypium hirsutum*) in (RA × T582)F*_2_*.

Combination	No. Plants	Expected Ratio	Expected Frequency
Red anther, normal branch	172	9	144
Cream anther, normal branch	25	3	48
Red anther, cluster	3	3	48
Cream anther, cluster	56	1	16
Chi-square			158.65 **

** *p* < 0.01.

## Data Availability

Data are contained within this article.
